# Therapeutic Potentials of Jamun (*Syzygium cumini*) and Its Integration Into Modern Food Technologies: A Review

**DOI:** 10.1155/ijfo/8197889

**Published:** 2025-08-10

**Authors:** B. S. Adithya, Mohammed Nayeem, Narashans Alok Sagar, Sourabh Kumar

**Affiliations:** ^1^Department of Food Technology and Nutrition, School of Agriculture, Lovely Professional University, Phagwara, Punjab, India; ^2^Department of Food Science and Technology, National Institute of Food Technology Entrepreneurship and Management, Sonipat, Haryana, India; ^3^Department of Biotechnology, University Institute of Biotechnology, Chandigarh University, Mohali, Punjab, India; ^4^University Centre for Research and Development, Chandigarh University, Mohali, Punjab, India; ^5^Department of Post Harvest Process and Food Engineering, Govind Ballabh Pant University of Agriculture and Technology, Pantnagar, Uttarakhand, India

**Keywords:** bioactive composition, extraction techniques, food application, jamun, nutraceutical

## Abstract

In a world where consumer preferences continue to pivot toward healthier and eco-conscious choices, jamun (*Syzygium cumini*) emerges as an underutilized resource with untapped potential. Despite the abundance of phytochemicals, the fruit also encounters postharvest losses, pointing toward extensive processing and additional research for sustainable utilization of food. The review brings together an understanding of existing knowledge on the therapeutic potentials of jamun and its integration into modern food technologies, providing valuable insights for food researchers and healthcare professionals aiming to leverage the health-promoting properties of this remarkable fruit to enhance human well-being. Different extraction techniques, including both traditional and eco-friendly methods, have been studied, demonstrating efficient ways to extract various bioactive compounds from jamun fruit, such as polyphenols, flavonoids, and terpenes. These compounds show antidiabetic, anticancer, anti-inflammatory, and antimicrobial properties, collectively enhancing the health-promoting properties of jamun. The versatility of jamun finds expression across multiple food sectors, spanning from producing wines and bakery items to antioxidant-rich popsicles aligned with contemporary health and sustainability trends which has been discussed. Further research must focus on the utilization of jamun pomace rich in fibers and antioxidants for sustainable food production.

**Summary**

• Jamun was found to be a rich source of bioactive compounds such as phenols, anthocyanins (Acns), terpenes, and carotenoids.
• Bioactive compound present in jamun exhibits pharmacological characteristics such as antioxidant, antidiabetic, anticancer, and anti-inflammatory activity.
• Green processing techniques were found to be a better alternative to conventional extraction techniques for the extraction of bioactive compounds.
• Incorporation of jamun extract or derivatives in innovative food products improved the nutritional profile of the final products.

## 1. Introduction

The native evergreen tree of jamun, recognized as Indian blackberry, jambola, or java plum, belongs to the Myrtaceae plant family and ranks as the world's second most phytonutrient-rich fruit globally [1, 2]. For the cultivation of jamun, clayey loamy soil was considered to be the best, and such soil type was commonly found in the Indian subcontinent and adjoining Southeast Asian countries such as Sri Lanka, Nepal, Malaysia, and Indonesia [3, 4]. Jamun cultivation undergoes a fascinating journey from budding as a flower (March to April months) which later on (May to June months) turns into fruit bodies with a greenish color and, at the final stage of maturity, turns into oblong/ovoid/round shape, single-seeded berries with deep pink/black color [5, 6]. The market predominantly offers two varieties of jamun, that is, “Ra” and “Kaatha” in which the Ra variety of jamun is much bigger and has a sweet aftertaste, whereas the “Kaatha” variety of jamun is much smaller in size and is acidic in taste. Genotype studies of jamun identified considerable differences in characteristics like fruit weight, diameter, pulp weight, and shape. Among 23 genotypes, maximum fruit weight (15.67 g), diameter (2.68 cm), and pulp weight (11.83 g) were observed in genotype KJS-300, maximum length-to-diameter ratio (1.70) indicating cylindrical shape in KJS-18, maximum canopy spread (15.75 m) in KJS-09, and minimum (6.65 m) in KJS-21 and KJS-43. Moreover, a study on accessions of jamun revealed differences in pulp percentage (49%–92%), TSS (total soluble solids) (10.8%–20.3%), titratable acidity (0.64%–1.69%), and phenolic content (23.00–318.6 mg GAE/100 g). The results are useful for germplasm development, breeding, and cultivar selection with characteristics such as seedless and increased fruit size [7, 8]. The fruit is considered a therapeutic food item due to its abundance in phytonutrients like myricetin, gallic acid, quercetin, chlorogenic acid, Acn, ferulic acid, catechin, malic acid, and caffeic acid [9, 10]. Acns in jamun give the fruit its distinctive black/bluish color and offer a range of health advantages, including anticancer, control inflammation, and neuroprotective properties. Jamun contains over 30 volatile flavor compounds, including trans and cis-ocimene, *α*-terpineol, and *β*-myrcene. The key esters contributing to jamun's characteristic flavor are hydrocarbyl acetate, geranyl butyrate, and terpinyl valerate [11]. Additionally, jamun juice is a significant source of essential minerals like calcium, magnesium, and potassium, and jamun peels are identified as a valuable source of flavanols, including myricetin 3-o-glucoside, syringetin, and laricitrin 3-o-glucoside [12]. Additionally, jamun leaves contain a range of bioactive compounds like alkaloids, terpenoids, myricetin, kaempferol, betulinic acid, n-nonacosane, n-triacontanol, terpineol, and myricetin [3].  Table 1 comprises the nutritional composition of jamun and its seeds. The seed extract of jamun has been noted to contain ellagic acid, ellagic tannins, quercetin, jambosine, and glycosides. These seed-derived glycosides inhibit the starch-to-sugar conversion and help in diabetes management. Moreover, ellagic acid and glucosidase present in jamun effectively overcome neurological problems, obesity, and kidney stones [18]. The juice of the fruit has been identified for its potent bactericidal properties against various pathogens, including *Salmonella typhimurium*, *Staphylococcus aureus*, and *Shigella flexneri* [2, 19, 20]. Moreover, studies have highlighted the efficacy of biosurfactant application, either alone or combined with xanthan gum, as an edible coating for jamun, enhancing its shelf life and quality. Notably, biosurfactant-treated fruits had better microbial protection throughout storage, thereby extending jamun's shelf life during postharvest handling [21]. Additionally, the utilization of modified atmospheric packaging has emerged as a pivotal technique in prolonging the shelf life of fresh jamun under cold and refrigerated conditions, allowing for preservation for up to 30 days [22].

Traditionally, jamun has been used in many traditional medicine systems like Ayurveda, Homeopathy, and Unani for treating diabetes, and various other ailments have been well documented by researchers. The Unani Pharmacopoeia of India serves as an authoritative reference for the preparation and classification of Unani medicines [23]. The WHO (World Health Organization) has also recommended the use of traditional medicine for diabetes treatment. Jamun is one of the medicinal plants that could be used for therapeutic purposes in diabetes mellitus, diarrhea, and loss of appetite. Both the leaves and fruits of black jamun possess notable medicinal properties. The fruit pulp and seeds are characterized by their sweet, acidic, and sour flavors, as well as their tonic and cooling effects. Traditionally, they are utilized in the treatment of various ailments, including diabetes, diarrhea, and ringworm [14]. Phytochemicals present in jamun, such as quercetin, gallic acid, and oleanolic acid, demonstrated remarkable efficacy, achieving over 90% cell cytotoxicity at concentrations of 2.5 *μ*g/mL and higher, while oleanolic acid showed moderate effectiveness at concentrations up to 5 *μ*g/mL [24]. It was also stated that oral administration of 4.5 g jamun seed powder capsules over a 12-week period exhibited a notable decrease in fasting plasma glucose levels, dropping from 105.67 to 96.03 mg/dL, with further reduction to 92.13 mg/dL by the end of the monitoring period. Additionally, postprandial blood sugar levels showed improvement, decreasing from 130.33 ± 3.44 to 118.83 ± 2.65 mg/dL after 28 days of oral administration, controlling glucose levels in the body [25].

## 2. Bioactive Composition of Jamun

### 2.1. Polyphenols

Phenols, prominent secondary metabolites in plants, constitute an aromatic compound featuring one or more hydroxyl (OH) groups, profoundly influencing the sensory attributes of colorful fruits and their derivatives [26]. Jamun, known for its bioactive compounds, encompasses various phenolic compounds, including flavonoids (such as Acn), flavanols (quercetin, myricitrin), and flavonols (catechins) [1, 27]. The aqueous extract of jamun yields a spectrum of phenolic acids, including tannic acid, gallic acid, chlorogenic acid, ellagic acid, caffeic acid, and p-coumaric acids characterized as organic carboxylic acids with a phenolic ring carrying either the C6-C1 structure of p-hydroxybenzoic acid or the C6-C3 arrangement of hydroxycinnamic acid, which play pivotal roles in conferring health benefits associated with jamun [6, 15]. The bioactive compounds, acting as active hydrogen atoms or electron donors, elevate the antioxidant potential of food products, contingent upon factors like cellular molecular targets and bioavailability. The integration of phenol-rich jamun extracts into food products could be used as an opportunity in the future to counteract lipid free radicals or impede radical formation, thereby curbing the generation of volatile products (aldehydes and ketones), mitigating unpleasant odors and averting lipid rancidity in food matrices [13, 26]. The comprehensive array of phenols present in jamun holds promise not only for augmenting the health benefits associated with its consumption but also for enhancing food product stability and quality through antioxidant effects and modulation of the oxidative process.

### 2.2. Flavonoids

**Figure 1:**
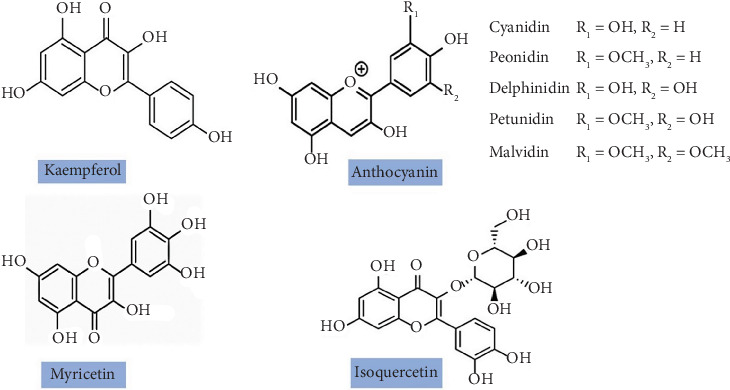
Structure of flavonoids present in jamun.

Flavonoids are diphenyl propane skeletal structures made up of two benzene rings attached to a pyrene ring, and the chemical formula for the structure is C_6_-C_3_-C_6_ [28]. Based on the oxidation of the central carbon atom, flavonoids are further divided into flavonols, flavan-3-ol, isoflavones, and Acns. Major flavonoids (Figure 1) present in jamun are procyanidin B1, malvidin, kaempferol, isoquercetin, petunidin, ricetin, rhamnose, flavanonols (dihydromyricetin, dimethyl-dihydromyricetin di-glucoside, and methyl- dihydromyricetin di-glucoside), Acn, catechin, and monoglucosides [15]. Following digestion, flavonoids absorbed in the small intestine undergo metabolism by Phase II enzymes. The absorption of the remaining flavonoids occurs in the large intestine, where colon microflora degrade conjugated metabolites, converting them into readily absorbable forms like phenolic acids [29]. Flavonoids are also reported to have significant antioxidant activity (AA), potentially attributed to the conjugation of the C2 double bond with the 4-oxo in A and C rings, as well as the presence of an O-dihydroxy group in the ring [9, 10].

### 2.3. Acns

Acns are water-soluble pigments with strong antioxidant and anticancer properties that belong to the flavonoid family and represent a diverse group of glycosylated compounds characterized by polyhydroxy and polymethoxy derivatives derived from flavylium cation. The sugars commonly bound to anthocyanidins are glucose, arabinose, and galactose, and depending on the conjugated bond present, Acn exists in red, blue, and purple colors. When the pH is between 1 and 3, red-colored flavylium cations are more stable. But when there is a loss of protons at high pH conditions, blue-colored quinonoid structures are formed; further, at pH 4–5, colorless hemiketals are formed [30], and the color change in Acn with variation in pH condition could be utilized in intelligent packaging of food products or could be used as a biocolorant. Further, based on structural variation in methoxylation and hydroxylation around benzene rings, Acns exist in six different glycoside forms, namely, cyanidin, peonidin, pelargonidin, delphinidin, petunidin, and malvidin [31, 32].

**Figure 2:**
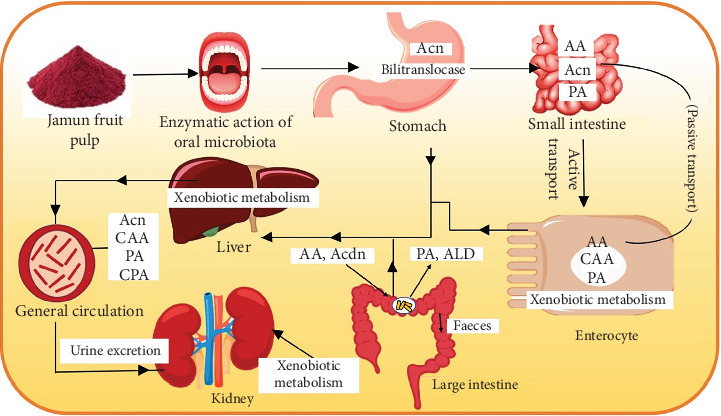
Representation of Acn absorption and metabolism pathway in the human body. AA: anthocyanidin, CAA: conjugated anthocyanidin, Acn: anthocyanin, PA: phenolic acid, ALD: aldehyde, CPA: conjugated phenolic acid.

The metabolism of Acn in the human body starts when Acn undergoes dynamic structural alterations within the oral cavity due to diverse pH and temperature conditions encountered postconsumption (Figure 2). Moreover, the presence of salivary amylase and the diverse oral microbiota contribute significantly to the deglycosylation process by exhibiting beta-glucosidase activity [33]. Salivary amylase also promotes partial degradation of Acn, which contributes to increased bioavailability [34]. *Stomach:* Bilitranslocase and glucose transporters help to transfer Acn in the stomach, and the acute effect of bilitranslocase helps in better circulation of Acn in the stomach. Under several studies, it was found that the interaction between gastric motility and pH plays an important role in the fate of Acn present in consumed food with gastric contents. The gastric environment, characterized by a pH range of 1.5–4 due to the influx of natural secretions such as hydrochloric acid and also enzymes present in the stomach, significantly affects the stability of Acn. *Small intestine:* One major mechanism involved in Acn movement across the small intestine is sodium-dependent glucose cotransporter [32]. It was reported that due to the deglycosylation process, Acn gets converted into anthocyanidins and enters epithelial cells by passive transport. The deglycosylation process in the gastrointestinal tract involves the enzymatic action of *β*-glucosidase and lactase phloridzin hydrolase present within the border of epithelial cells [35]. *Enterocyte*: Upon traversing the enterocyte walls, Acn undergoes xenobiotic metabolism [34] which leads to the modification that augments its hydrophilicity and subsequent elimination route via bile and urine. The xenobiotic metabolism interaction comprises two phases: Phase 1 encompasses oxidation, reduction, and hydrolysis reactions, while Phase II involves conjugation reactions. *Large intestine:* The large intestine offers a considerable surface area for the absorption of Acns. Once Acn reaches the intestine, it undergoes a sequence of transformative reactions catalyzed by Phase II enzymes, such as methylation and sulfation. These reactions lead to the presence of methylated and sulfated Acn in human plasma and urine. Moreover, before conjugation, AA undergoes the degradation of phenolic acids and aldehydes in the intestine [36]. Additionally, flavonoids, including Acn, undergo extensive metabolism by colonic microbiota, leading to their absorption as simpler phenolic acids and aldehydes [35]. *Gut:* In the gut, Acns are degraded by various metabolisms such as deglycosylation, C ring cleavage, and decarboxylation, and the metabolites are phenyl aldehydes, benzaldehyde, hydroxybenzoic acid, and hippuric acid [33, 37]. Metabolites arising from Acn metabolism, particularly those stemming from microbial processing, emerge as predominant forms in circulation. These metabolites have potential significance in imparting various health benefits linked to the consumption of Acn-rich dietary sources.

### 2.4. Terpenes

**Figure 3:**
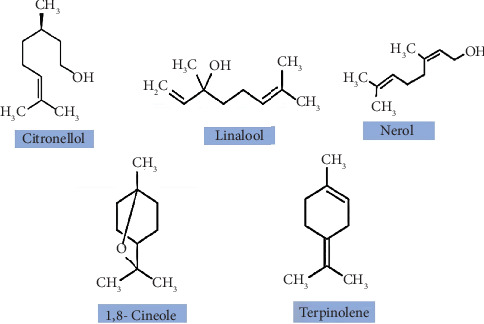
Structure of terpenoids present in jamun.

Terpenes, composed of multiple units of isoprene, are organic compounds renowned in the food industry for their distinctive aroma. In jamun, terpenoids (Figure 3), such as oleanolic acid, *β*-sitosterol, and betulinic acid, along with monoterpenoids like *α*-terpineol, 1,8-cineole, mysterol, terpinolene, and *β*-pinene, are prevalent [6]. These compounds offer a range of health benefits, including anti-inflammatory, antihyperglycemic, and antimicrobial properties, thereby aiding in the prevention of fungal and bacterial infections [28].

### 2.5. Carotenoids

Carotenoids are natural pigments synthesized from eight isoprene units, categorized into cyclic and linear hydrocarbons. The characteristic yellow hue of these compounds is attributed to their conjugated C-C double bonds of polyene chain. Jamun, a fruit rich in carotenoids, contains between 48 and 55 mg/100 g of these compounds, including *β*-carotene, zeaxanthin, *β*-cryptoxanthin, and lycopene. Research has identified all-trans-lutein (43.7%) and all-trans-*β*-carotene (25.4%) as the predominant carotenoids in jamun pulp [38]. Known for their antioxidant properties, carotenoids like phenol contribute to eye health and possess antiaging effects [6].

## 3. Nutraceutical Properties of Jamun

Jamun is widely used because of its therapeutic and nutraceutical health benefits and was found to have better control over oxidative stress, hyperglycemia, inflammation, and cancer and was also found to have antimicrobial activity. Flavonoids exhibit AA by neutralizing free radicals through hydrogen or electron transfer, largely due to the presence of hydroxyl groups in their structure. This antioxidant mechanism allows flavonoids to effectively scavenge reactive oxygen species (ROS), singlet oxygen, and various free radicals, which helps in preventing diseases associated with oxidative stress, such as cancer, neurological disorders, and immune dysfunction [39]. Lead is a heavy metal that is toxic and has been shown to damage several body systems, including the kidneys. In a Wistar rat study, exposure to lead at a dosage of 50 mg/kg body weight raised serum creatinine (from baseline to high levels on Days 7 and 14), urea, and uric acid—all important markers of kidney injury. Contrarily, rats given jamun seed extract at a dose of 200 mg/kg body weight exhibited significant decreases in these biomarkers. There were no changes in kidney biomarkers from the seed extract treatments, which support the safety profile of the extracts and provide protection against lead-caused renal toxicity by normalizing kidney biomarkers [40]. One of the well-known approaches to treating obesity includes the inhibition of pancreatic lipase by plant-based natural substances. In a study, jamun seeds were investigated in white albino Wistar rats induced to be obese by a high-fat diet (aged 8–9 weeks, weighing around 110 g). Ethanolic seed extract exhibited 103.79 mg GAE/g of TPC (total phenolic content), whereas IC_50_ values for the ethanol extracts and standard butylated hydroxytoluene (BHT) were 93.03 and 10.84 *μ*g/mL, respectively. The antiobesity activity of the extracts was equivalent to that of orlistat at 10 mg/kg body weight. Orlistat and jamun seed at a concentration of 0.1 and 1.0 mg/mL inhibited 95.71% and 60.84% inhibition of lipase activity, respectively [41]. Aqueous extract of jamun pulp was reportedly highly active against the etiological agent *Bacillus anthracis* causing gastrointestinal anthrax. The extract reduced the count of bacteria by 6 logs within 2 h at ≥ 1.9% *w*/*v* concentration and remained highly effective even when heated. The aqueous portion was not only compatible with FDA-approved anthrax antibiotics but was also nontoxic to useful gut bacteria. It also demonstrated a long-lasting bactericidal effect within a 16-mm zone of inhibition for 72 h [42]. The detailed mechanism of the nutraceutical properties of jamun is mentioned below.

### 3.1. Antidiabetic Potential

**Figure 4:**
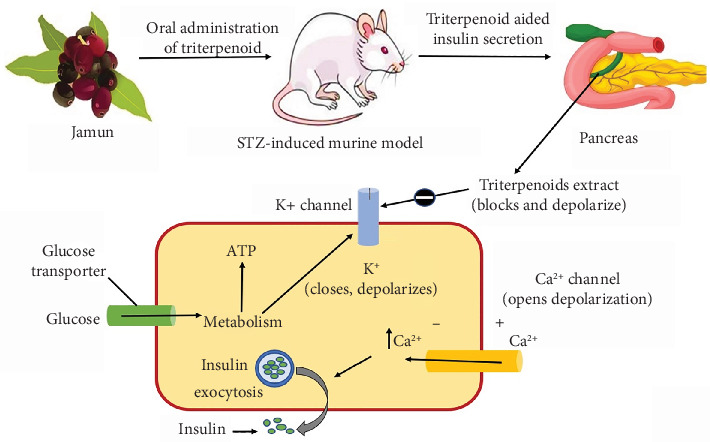
Terpenoid-assisted regulation of insulin secretion in pancreatic beta cells.

Diabetes, a chronic disease caused by increased hyperglycemic levels in the blood, might be due to the unavailability or inefficiency of the human body to utilize insulin effectively [43]. *α*-Amylase, a digestive enzyme that hydrolyzes starch to glucose, is responsible for the increase in postprandial hyperglycemia levels in the human body. Bioactive compounds present in jamun seeds, such as glycosides, jambosine, alkaloids, and polyphenolic compounds like quercetin, catechins, and tannins present in fruit parts, help to inhibit *α*-amylase activity [44]. Among these bioactive compounds, one major bioactive compound in jamun is triterpenoid, which consists of various health benefits such as better insulin secretion, inhibition of *α*-glucosidase, aiding in *β*-cell regeneration, and good AA. Stimulated insulin release is a complex process crucial for maintaining glucose homeostasis in the body. One pivotal mechanism underlying this process involves the interplay of various cellular events, as illustrated in Figure 4. Insulin release from pancreatic beta cells could be intricately regulated by glucose levels and also with the help of pharmacological jamun extract agents such as triterpenoid. Under resting conditions with low ATP levels, potassium ions (k^+^) traverse ATP-gated potassium channels, preserving the intracellular potential at a polarized, negative state, which minimizes insulin release. Elevated glucose levels stimulate ATP production, leading to increased insulin secretion through the closure of potassium channels and subsequent cell depolarization. This depolarization event prompts the opening of voltage-gated calcium channels (Ca2+), allowing calcium ions to enter the cells. The subsequent rise in intracellular calcium concentration triggers the secretion of insulin, a pivotal hormone in blood sugar regulation [45, 46]. In this process, triterpenoid extract from jamun could be classified as insulin secretagogues because it helps in closing the ATP-dependent potassium channels, including membrane depolarization, and subsequently enhancing insulin release. Understanding the intricacies of these regulatory pathways gives valuable insights into the therapeutic inventions aimed at modulating insulin secretion [47, 48]. It was also stated that oral administration of terpenoid-rich jamun fruit extract reduced fasting hyperglycemia by 27.4%, and a notable increase in fasting insulin level (42%) was also observed in treated groups in comparison to streptozotocin, an antibiotic derived from *Streptomyces achromogenes* that selectively targets pancreatic cells and negatively affects insulin synthesis, induced diabetic group [48]. Terpenoid-rich extract also reportedly increased phosphorylation of protein kinase by twofold and glucose transporter 4 expression by 1.5-folds in skeletal muscles, indicating increased insulin sensitivity [47].

Polar solvents such as methanol and n-butanol were found to have better efficiency in *α*-glucosidase inhibition than nonpolar solvents (ethyl acetate). As shown in Table 2, the IC_50_ (concentration of fruit part required to inhibit pancreatic *α*-amylase activity) value of jamun extract was much lower than that of standard acarbose (control diabetes) indicating jamun to be a better alternative to the commercially available medical drugs. Several in vitro and in vivo studies confirm the potential of jamun extract in controlling diabetes. In this regard, jamun seed, the underutilized fruit part, was found to have better antidiabetic properties than fruit juice [50] whereas mice treated with a 14.36% jamun seed extract–infused diet showed a better reduction in hyperglycemia than a diet prepared with 12.29% fruit pulp extract. A similar study stated that [52] jamun pulp and kernel portion inhibited *α*-amylase activity by 53.8% and 98.2%. Bioactive compounds, catechin and gallic acid, present in jamun-infused ice pops also reportedly inhibited 60% *α*-amylase activity [2]. The inhibition rate is mostly dose-dependent, as studied by [59] where in jamun-based confectionery, percentage inhibition increased from 18.42% to 55% with an increase in jamun concentration from 20.13 to 100 mg. The phenols found in jamun exhibit inhibition of *α*-amylase through a noncompetitive mechanism, and these compounds bind to a site distinct from the active site of *α*-amylase, thereby hindering the interaction between starch and the enzyme's active site, ultimately leading to the suppression of glucose production.

### 3.2. Antioxidant Properties of Jamun

Across all life forms, a delicate equilibrium is maintained between the generation and degradation of ROS. Any disruption in this balance, such as an imbalance between antioxidants and ROS, can induce oxidative stress, which poses significant health risks [60], and to overcome such problems, synthetic antioxidants such as butylated hydroxyl anisole, propyl gallate, and butylated hydroxyl toluene were used; however, lately, these antioxidants were found to have a carcinogenic effect, which initiated the need for plant-based antioxidants with endogenous protective systems [16] and one such plant-based antioxidant-rich fruit source is jamun. Jamun extracts can scavenge free radicals produced as a result of exposure to ionizing radiation, and the percentage inhibition rate was found proportional to the polyphenolic composition of jamun [14]. The AA of gallic acid present in jamun might be due to the presence of three hydroxyl groups, and for monophenols such as sinapic acids, antioxidants might be due to the presence of one or two methoxy substitutions in ortho and OH positions [9, 10]. The AA of the fruit part has been discussed in Table 2, but the underutilized seed portions were also reported to have inhibition between 73.8% and 92.4%, which might be due to the presence of kernel fractions attached to the seeds [52]. A similar study showed that 0.2 mg/mL jamun seed extract exhibited 74.27% scavenging activity, and the activity increased in a dose-dependent manner [16]. Moreover, an in vivo investigation revealed that incorporating 1.5% jamun leaf extract into the diet of snubnose pompano (*Trachinotus blochii*) fish afflicted with *Vibrio parahaemolyticus* bolstered their defense mechanisms, resulting in an impressive 81.48% increase in survival rate [60].

### 3.3. Anticancer Activity of Jamun

Cancer, one of the life-threatening diseases, might be associated with numerous factors such as changing lifestyle, genetic, and hormonal changes. Cancer is induced by a multiple-stage process involving the conversion of normal cells to tumor cells, and this process progresses from a precancerous lesion to a malignant tumor. Exposure of animals to stress conditions induces a rapid decrease in oxygen levels, triggering oxidative reactions in aerobic metabolism that generate ROS such as hydrogen peroxide, singlet oxygen, superoxide, and hydroxyl radicals, collectively known as free radicals [60]. The WHO's 2020 report highlights lung cancer (1.80 million deaths), colon and rectum cancer (916,000 deaths), and breast cancer (685,000 deaths) as the most prevalent types of cancer leading to mortality globally (Cancer (who.int)). For cancer treatment, several antineoplastic drugs such as vincristine, Taxol, and docetaxel have been used, but these drugs possess toxic effects on normal cells, and to solve the problem, researchers are now focusing on medicinal plant extracts [54]. A hallmark of the therapeutic drug is its ability to induce apoptosis because apoptosis is considered a barrier to tumor growth [53]. Acn present in jamun helps to metabolize the enzymes, regulate gene expression, and modulate cell proliferation and apoptosis. It was reported that the inclusion of a jamun powder–enriched diet not only improved tumor latency by 2 weeks but also reduced tumor incidences and tumor multiplicity by 65% and 1.8 tumors/min [54]. It was stated that jamun pulp extract (40 *μ*g/mL) rich in Acn, flavonoids, and stilbenoids suppressed colon cancer cell proliferation in mice by 60%, and in a similar study, 2 mg/mL of jamun extract inhibited the viability of lung carcinoma cells by 80% [53]. The antioxidants present in jamun might have decreased ROS, thus preventing DNA damage and the spreading of cancer. All these studies prove jamun extract to be a promising alternative to synthetic drugs for chemotherapy [55]. In a study, environmentally friendly gold nanoparticles developed from flavonoid and terpenoid-rich jamun leaf extract attributed to the significant AA, as evidenced by 91.56% DPPH (2,2-diphenyl-1-picrylhydrazyl) and ABTS (76.59%) tests, have exceptional anticancer potential [61]. Furthermore, jamun extract–infused golden nanoparticles showed tyrosinase inhibition (31.04% at 0.1 mg/mL), which raises the possibility that they regulate enzymatic pathways linked to cancer. Because the nanoparticles target oxidative stress and tumor development processes, their strong antioxidant and enzyme inhibitory actions suggest that they may find use in cancer therapy. Crude jamun seed extract extracted by the decoction method also exhibited strong anticarcinogenic activity against proliferative carcinomas. Among the solvent extracts, the methanolic extract showed the strongest anticancer activity against acetone and ethanolic extract. At a concentration of 125 *μ*g/mL, the methanolic, acetone, and ethanolic extracts inhibited 49.57%, 35.01%, and 27.67% of carcinoma cells, respectively. The acetone extract at 31.25 *μ*g/mL and the ethanol extract at 125 *μ*g/mL also inhibited carcinoma cell viability by 28.11% and 27.67%, respectively [62]. Hydroalcoholic fruit pulp and juice extracts reportedly showed strong cytotoxic activities against breast cancer cells with an IC_50_ value of 80.75 *μ*g/mL [63].

### 3.4. Anti-Inflammation Property of Jamun

Inflammation arises from a dysregulated response to both exogenous and endogenous antigens, resulting in the accumulation of proinflammatory cytokines and associated downstream signals. This inflammatory process can give rise to various health issues, including respiratory challenges, dermatological conditions, and neurodegenerative disorders such as Parkinson's and Alzheimer's disease. To manage these conditions, synthetic medications like nonsteroidal anti-inflammatory drugs and corticosteroids are commonly prescribed. However, long-term usage of such drugs leads to problems such as an upset digestive system and liver and gastric lesions [51]. Edema paw is a classic example of a cardinal sign of acute inflammation and is an important tool for evaluating the anti-inflammatory properties of any food products, whereas the myeloperoxidase enzyme present in neutrophils, monocytes, and macrophages is reported to have a direct effect on the neutrophil concentration in inflamed tissues, which helps to judge neutrophil infiltration in the inflammatory process [64, 65]. Inflammation in carrageenan-induced mice might be due to the release of compounds such as prostaglandins and histamine, and prostaglandins were found to be an important cause of intense pain and fever. In a study, it was reported that the crude methanolic extract of jamun followed by ethyl acetate and dichloromethane jamun fractions resulted in 86.11%, 67.09%, and 63.23% inhibition in carrageenan-induced inflammation in mice, which might be related to the ability of crude jamun extract to block cyclo-oxygenase, which in turn stops the production of prostaglandins [66]. Jamun leaf essential oil extract (3–30 mg/kg) rich in kaempferol also attenuated edema paw in carrageenan-induced mice from 38 to 18 mg/kg and reduced myeloperoxidase enzyme activity from 31.58 to 7.38 units/g paw tissue, and with a reduction in edema paw, jamun extract exhibited anti-inflammatory activity by suppression of tumor necrosis factor *α* by 422.91–112.07/mL of tissue exudate [67]. Further, it was stated that jamun leaf extract rich in myricetin, triterpenoids, and acetyl oleanolic acid concentration helps acetylcholinesterase enzymes to break acetylcholine, an anti-inflammatory molecule, into choline and acetate, thus preventing tissue damage generated by inflammation [60].

### 3.5. Antimicrobial Activity of Jamun

Antimicrobials such as antibiotics, antivirals, antifungus, and antiparasites are compounds that are used to suppress/inhibit the growth of harmful microorganisms present in plants and animals. But misuse and overdosage of drugs have paved the way for pathogens to develop resistance against drugs and also developed a defense mechanism known as biofilm (a complex matrix of microorganisms made up of polysaccharides, proteins, and organic compounds that bind with the cells and strongly attach to the abiotic and abiotic surface) [68]. To prevent such antimicrobial resistance, jamun-based plant extracts are utilized as antibacterial agents which inhibit biofilm by reducing colonization of surface and epithelial mucosa by microorganisms and prevent the spreading of disease. The WHO has identified antimicrobial resistance as one of the top 10 global public health threats. Among the myriad of challenges posed by antimicrobial resistance is the prevalence of pathogenic parasites such as *Leishmania amazonensis*. This parasite belongs to the Trypanosomatidae family and encompasses more than 20 protozoa species. Understanding and addressing the threats posed by such pathogens is crucial for global health efforts to combat infectious diseases [58] and the drugs used to cure the infection are toxic in nature, whereas essential oil extract *α*-pinene from jamun leaf extract exhibited a better inhibitory effect against intracellular and axenic amastigotes (IC_50_ = 15.6 and 16.1 *μ*g/mL) and the extract also emerged as a safer option for macrophages, exhibiting significant SI (number of times the extract having a toxic effect on the parasite than on the mammalian cell) of 21.5, 26.4, and 27.2 for promastigotes, axenic amastigotes, and intramacrophage amastigotes. An SI value below 1 indicated it to be more toxic to macrophages than to the parasite. Jamun leaf extract could also be utilized as an alternative to chemical disinfectants. Chemical disinfectants can have adverse effects on human health and lead to the development of disinfectant-resistant strains and antibiotic resistance. The leaf extract exhibited antimicrobial effect against pathogenic strains *S. aureus* and *Pseudomonas aeruginosa*, as reported by [69]. The minimum inhibitory concentrations (MICs) for *S. aureus* and *Pseudomonas aeruginosa* were 625 and 1250 *μ*g/mL, respectively. The extract was also determined to be nontoxic, with an IC_50_ of 320 *μ*g/mL, which shows that it could be a safe and effective natural disinfectant. In a recent study, methanolic extract of jamun seeds exhibited significant antibacterial activity by giving an inhibition zone of 19.00 mm against *Staphylococcus epidermidis* in comparison to antibiotic gentamicin, which gave an inhibition zone of 18.33 mm. The extract also showed the lowest MIC (0.32 mg/mL) and minimum bactericidal concentration (MBC) values (0.52 mg/mL) against the bacterial strain. Overall, the methanolic extract effectively outperformed gentamicin in both inhibition zone and bactericidal potency [39].

The extract has also been used for metallic nanoparticles' biogenesis due to the cost-effective and nontoxic nature. In the study reported by Das [61], gold nanoparticles synthesized using an aqueous extract of jamun leaves served as a stabilizing and capping agent. In comparison with streptomycin, with an inhibition level of 10.55–16.24 mm, the nanoparticles biosynthesized with jamun extract exhibited vigorous antibacterial properties with inhibition ranges of 11.02–14.12 mm. The promising antibacterial characteristics of the nanoparticles depict their potential as antimicrobial fighters. Jamun extract also demonstrated significant antimicrobial activity against both gram-positive and gram-negative bacteria, potentially attributed to synergistic interactions among its phenolic compounds. The MIC and zone of inhibition against various pathogens range from 0.5 to 2.5 mg/mL and 14.3–23.0 mm, respectively. Notably, *S. aureus* exhibits the highest sensitivity to jamun extract, with a zone of inhibition of 23.0 mm [9, 10]. Similarly, a study by [11] revealed that essential oil isolates from jamun leaves, such as 7-hydroxycalamene, 7-acetoxycalamenene, and 1-epi-cubenol, exhibit potent antifungal activity against *Ralstonia solanacearum* and *Choanephora cucurbitarum*, outperforming standard *α*-pinene (Table 2). These findings underscore the potential of jamun-derived compounds as effective antimicrobial agents with broad-spectrum activity against various pathogens. Jamun also found useful tools in the food sector, such as aquaculture marketing. In aquaculture, microorganisms could also lead to a large sum of economic loss in fishery due to an increased mortality rate caused by bacterial and viral infections. However, a study [3] studied that oral administration of a 1% jamun extract–infused diet to Pacific white leg shrimp increased superoxide dismutase and catalase activity (enzymatic antioxidants). Superoxide dismutase scavenges superoxide radicals and protects against oxidative degradation of tissues from 87.38 to 93.51, whereas catalase converts hydrogen peroxide to water and oxygen and prevents oxidative damage caused by ROS in the pancreas from 12.42 to 15.31 and has a 10% higher survival rate in comparison to the control specimen after postchallenge. The favorable increase in superoxide activity is linked with increased host immunity. The diet also helped to reduce aspartate aminotransferase and alanine aminotransferase levels by 5.25 and 14.55, which implies reduced stress conditions in shrimp leading to a decline in ROS production. It was also reported that jamun-based antimicrobial compounds are a rich source of secondary metabolites such as flavonoids and polyphenols. These bioactive compounds have attained a much better stability rate in comparison to a range of commercially available antibiotics [68]. It was stated that oral administration of 4.5 g jamun seed powder capsules over a 12-week period exhibited a notable decrease in fasting plasma glucose levels, dropping from 105.67 to 96.03 mg/dL, with further reduction to 92.13 mg/dL by the end of the monitoring period. Additionally, postprandial blood sugar levels showed improvement, decreasing from 130.33 ± 3.44 to 118.83 ± 2.65 mg/dL after 28 days of oral administration controlling blood glucose levels [25]. The incorporation of 2.59 and 2.74 g/kg jamun fruit extract into the diet of juvenile *Cyprinus carpio* exhibited superior weight gain, enhanced immune parameters, and improved antioxidant enzyme activity, ultimately leading to increased survival rates following exposure to *Aeromonas hydrophila*, highlighting its potential as a natural feed additive in aquaculture [70].

## 4. Extraction Techniques for Jamun Processing

**Figure 5:**
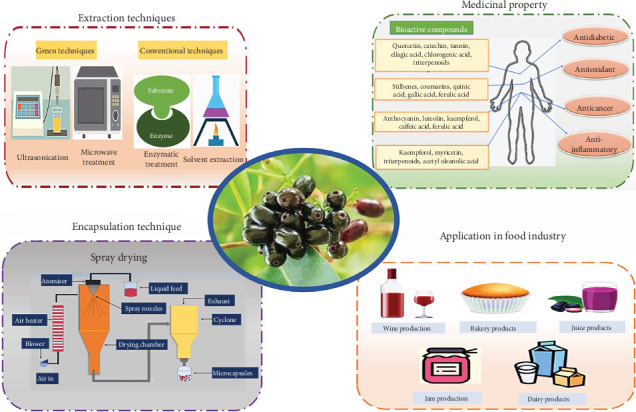
Extraction process of jamun juice and its utilization in jamun-based food products.

Jamun is an inexpensive source of bioactive compounds, and there is a vast scope in the food industry for the implementation of new processing techniques for the easy recovery of these bioactive compounds from jamun residues. Preservation of fruit juice by techniques such as pasteurization was cost-effective, but with extended processing time, volatile flavor and aromatic compounds of fruit juice tend to degrade bioactive compounds and organoleptic properties of the final product [71] and some conventional techniques such as enzymatic treatment and solvent extraction are labor-intensive and time-consuming, whereas green processing techniques such as ultrasonication (US), microwave (MV), and foam mat drying (FMD) were found to be more promising for the extraction of a diverse number of compounds from food products and increase final product yield. Green processing helps to overcome the drawbacks of conventional methods and ensures high-quality processed food products that are safe for consumption (Figure 5).

### 4.1. Conventional Extraction Techniques

#### 4.1.1. Enzymatic Treatment

Enzymes are the biological catalysts that speed up the rate of reaction in any food product. Pectinolytic enzymes, a heterogeneous group of enzymes, were commonly preferred for their ability to break down cell wall components, which significantly enhances juice extraction and improved recovery of bioactive compounds, thereby maximizing extraction efficiency and nutritional profile of the juice, aligning with consumer demand for healthier options [72]. During depectinization process, pectin present in the middle lamella of the cell wall breaks down and further pectin flocculates with the protein present in the juice which helps to reduce juice turbidity and it was reported that [73] addition of 0.05% *w*/*v* of pectinase enzyme (incubation time 75 at 40°C) from *Aspergillus aculeatus* strain slightly increased *a*^∗^ value of jamun juice from 2.65 to 3.25 and 83.88 NTU more reduction turbidity was reported in comparison with the control sample. Similar to jamun enzymatic treatment of blackcurrant juice with a pectinolytic enzyme at 45°C for 60 min led to notable increases in TPC, Acn, and AA with enhancements of 2.4, 2.8, and 2.7 times, respectively [72]. Tannase enzymes were also found effective in increasing quantity and quality of fruit juice as reported. Tannase from *Aspergillus ficcus* strain also effectively reduced jamun juice turbidity by 78.79% and increased juice yield by 14% in comparison to the control jamun juice [73]. Likewise, jamun juice treated using 0.05% tannase enzyme at 40°C for 80 min significantly enhanced the clarity of the juice by 42.39%, increased the TPC to 128.31 mg GAE/g, and boosted the juice yield to 79% [71].

#### 4.1.2. Solvent Extraction

Traditional methods of extracting bioactive compounds rely on organic solvents such as acetone, methanol, and ethanol, which, while effective, pose significant drawbacks, including high toxicity and environmental concerns. Given the polar nature of the compounds found in jamun, employing alternative solvents like pure water and acidified water presents a sustainable approach for extraction. This method not only reduces waste and minimizes the use of harmful reagents but also ensures the production of high-quality and safe extracts [74]. Solvent extraction has been commonly used for the extraction of phytochemicals from the jamun seed and leaves' portion. In the solvent extraction during the mass transfer process, an increase in the concentration gradient between solid and liquid tends to improve the diffusion rate, which in turn improves the solubility of the solvent in jamun extract, resulting in better juice yield [38]. It was also stated in a study that aqueous solvents significantly outperform their absolute counterparts in phenolic extraction, irrespective of extraction time and conditions. Among the aqueous solvents tested, aqueous acetone provided the highest TPC extraction at 111.9 mg, followed by aqueous methanol at 89.8 mg and aqueous ethanol at 83.6 mg, and water alone yielded a notable TPC of 78.1 mg from jamun seeds [75]. A similar study highlighted the effectiveness of using alternative solvents for extracting bioactive compounds from jamun. Jamun pulp treated with water at 75°C extracted 1347.27 mg GAE/100 g of TPC, whereas jamun pulp treated with 50% methanol extracted significantly higher TPC of 4019.39 mg GAE/100 g. Additionally, using water with 2.4% citric acid increased total monomeric Acn content by 209.31% compared to the fruit pulp. When water-based solvents are combined with citric acid or methanol, they act as highly efficient alternatives for extracting valuable compounds from jamun [74]. Ethanol–water mixtures demonstrate superior extraction efficiency for phenolic compounds compared to single-component solvent systems. The incorporation of water into organic solvents enhances the polarity of the extraction medium, which facilitates a more effective extraction of polyphenols. This polarity increase allows for better solubility and interaction between the solvent and phenolic compounds, leading to improved yield and extraction rates [76, 77]. It was also stated in a study that solvent extraction of jamun seeds at an optimum treatment condition with solid (S):liquid (L) ratio of 51.6:1 mL/g (water as solvent) for an incubation period of 49.2°C for 89.4 min increased total extraction yield, TPC, and AA by 17.3%, 415 mg GAE/g, and IC_50_ 35.4 *μ*g mL^−1^ [38]. Similarly, solvent extraction of jamun seed portion using 50% *v*/*v* ethanol for 45 min extracted 400.88 mg GAE/100 g TPC and 529.12 mg/100 g TFC (total flavonoid content) [78]. Sequential cold percolation was also found to be useful in enhancing the AA of jamun leaf extract. As reported, the DPPH scavenging activity of acetone and toluene jamun leaf extracts had IC_50_ values of 20 *μ*g/g and 1000 *μ*g/mL, respectively. The significant antioxidant effect of the acetone leaf extract might be due to the difference in polarity of the solvent used. While superoxide radical scavenging activity of ethyl acetate treated leaf extract was at its peak at IC_50_ 157 *μ*g/mL, the increase in AA might be due to the presence of hydroxyl group in the phenolic structure, promoting free radical scavenging activity [79].

### 4.2. Green Food Processing Techniques

#### 4.2.1. US

A significant challenge in the fruit and vegetable processing industry is the presence of spoilage microorganisms. Although these microbes do not pose a direct health risk to consumers, they can cause quality degradation, reduce the shelf life of products, and lead to substantial economic losses. Green processing technique US is gaining recognition as an innovative method for food preservation. The antimicrobial effects of ultrasound are primarily due to cavitation and the generation of free radicals. Cavitation leads to mechanical disruption of cell walls and membranes, while the intense conditions within the collapsing bubbles facilitate the formation of hydroxyl radicals [56, 80]. These radicals can damage microbial DNA and other cellular components, resulting in cell death. This dual mechanism underscores the effectiveness of ultrasound as a potent antimicrobial tool [81]. In addition, thermosonication has been reported to outperform pasteurization in terms of microbial inactivation and stability during storage in jamun dairy dessert. Thermosonication achieved a 1 log CFU/mL greater reduction in microbial counts and demonstrated enhanced stability over a 21-day period. It resulted in significantly lower counts of yeasts and molds, aerobic mesophilic bacteria, and lactic acid bacteria, with reductions of 3, 2, and 2.8 log CFU/mL, respectively [82]. Higher temperatures during US reduced solvent viscosity while increasing vapor pressure, allowing more solvent vapors to penetrate cell structures and intensify cavitation [76, 77]. In the US of jamun juice, the fruit part plays a significant role. With the increase in fruit concentration, surface tension and viscosity of juice also increase, which in turn reduces the cavitation efficiency. Sonication of jamun juice was also studied to have much better AA in comparison to some traditional processing techniques. In a comparative study, MV treatment at 400 W for 240 s and US at 70°C yielded 8.197 and 8.52 mg of C3GE/g and 40.63 and 47.33 mg of GAE/g for total Acn and TPC, respectively [83], indicating US to be a better fit for increased phytochemical extraction. Today, US is also combined with different processing techniques, and one such treatment combination is US-assisted vacuum treatment, where the US improves the osmotic dehydration of jamun pulp with the aid of vacuum pretreatment. With an increasing processing time of US-assisted vacuum treatment, the driving force between the hypertonic solution and the dilute part of jamun pulp also increased, which in turn increased moisture loss and solute uptake by 8.53–9.27 × 10^−10^ m^2^/s and 3.81–4.39 × 10^−10^ m^2^/s [56]. In conclusion, thermosonication stands out as an innovative approach in food preservation, combining ultrasound and heat to effectively maintain key nutrients while simultaneously reducing microbial threats [82].

#### 4.2.2. MV-Assisted Extraction

MV extraction stands out as an environmentally friendly process due to its reduced time requirements, minimal solvent use, and its ability to serve as a sustainable alternative to traditional extraction methods without emitting carbon dioxide. The application of MV power induces heating, leading to energy absorption and diffusion within the plant matrix. This process effectively breaks down cell walls, facilitating the release of bioactive compounds into the solvent, making it an efficient and green extraction method [84]. During MV treatment, electromagnetic waves penetrate deep inside the food, resulting in rapid vibration of water molecules, and the vibration effect was due to the friction generated by the dipole movement of water molecules by the force of attraction and repulsion, which further helps in the uniform heating of food products [42]. MV treatment was found to be an effective method in enhancing the bioactive composition of jamun juice, as reported in a study; after MV treatment, phenol, flavonoid, Acn, and DPPH activity increased in the range of 11.95–26.05 mg GAE/g, 5.46–11.17 mg QE/g, 2.78–5.87 mg CE/g, and 0.30–1.5 mg (AAE/g) [84]. Similarly, in a study, three distinct phytochemical extraction methods, namely, MV, shaking water bath extraction, and conventional extraction, were investigated to enhance the extraction efficiency of phytochemicals from jamun pomace, and it was reported that conventional extraction at 60°C for 90 min with a solid to liquid ratio of 1:30 and 0.05% HCl exhibited the highest extraction efficiencies of total phenols and flavonoids, whereas MV treatments with 80% MV amplitude for 2 min with a solid to liquid ratio of 1:30 and 0.05% HCl surpassed in antioxidant activities and total Acn content, respectively [42]. MVs, in combination with conventional processing techniques like hot air drying, could also effectively reduce the processing time required for jamun processing. It was stated that MVT of dried jamun powder treatment at 70°C resulted in maximum retention of phenol (31.52 mg GAE), Acn (11.99 mg malvidin glucoside), and AA (28.63 mg BHA/gm) [85]. MV processing is not only limited to the fruit part but also helps in better leaching out of polysaccharides from jamun seed, which might be due to better movement of biomolecules after the treatment or might be due to better solvent penetration into the plant matrix. It was reported that a polysaccharide content of 4.71% was extracted from jamun seeds by MV treatment at optimum conditions of S:L ratio of 1:15 g/mL, power 515 W, pH 3.2, and at a processing time of 3 min. Further, at a pH below 4, the condensation process converts the insoluble form of polysaccharides to a soluble form, and it in turn enhances extraction yield [86].

#### 4.2.3. FMD

This could also be used as an alternative to spray drying and freeze drying in jamun processing because they are cost-effective and require a smaller surface area. In FMD, the food product is mixed with a stabilizing agent to get a stable foam layer during different stages of drying. It was reported that in foam mat dehydrated jamun powder, Acn concentration varied from 1552 to 2298 mg/kg, and a slight decline in Acn content might be due to the presence of AA polymers such as procyanidins and phenolic acids, which at higher temperatures lead to oxidative degradation of Acn and flavonoids [87]. At the time of jamun processing, more than 20% of jamun seed is disposed of as food waste, and to prevent such wastage, phenolic extract from jamun seed and pomace was recently purified and concentrated using membrane processes such as ultrafiltration and nanofiltration. The rejection of phenolic compounds through the membrane is based on molecular weight. However, not all membrane processes are solely governed by molecular weight–based separation; many also function through mechanisms such as chemical affinity, electric charge interactions, and other physicochemical properties. At low molecular weight, heavy particles like cellulose and polysaccharides are rejected due to the smaller pore size. It was studied that ultrafiltration of jamun leaf extract operated at a low operating pressure of 276 and 424 transmembrane pressure difference with a 100-kDa membrane, recovering 59%–66.7% TPC with 49%–58.3% purity. One of the disadvantages of the microfiltration technique is the decrease in permeate flux, which was found to be directly proportional to the processing time. Pore blocking constant and cake layer constant for unfiltered and filtered jamun leaf extracts were 2.95 and 2.13 × 10^−3^ S^−1^ and 3.50 and 0.85 × 10^6^ Sm^−2^ [88]. The stability of jamun products was tested in powdered and liquid states under various storage conditions. Foam mat–dried jamun juice powder had good stability up to 150 days at 4°C, 25°C, and 35°C, with flavanol content unaffected and a 7%–9% decrease in Acns, mainly due to storage time but not temperature. TPC was retained at high levels, 96% after 35°C, and low water content (1.85%) prevented microbial proliferation. The incorporation of maltodextrin (MD) probably shielded the Acns from water and potential damage [89]. For comparison, a jamun pulp drink sweetened with stevia had decreased major quality factors, such as pH, viscosity, vitamin C, AA, and color—over 90 days' storage, with higher acidity and TSS, presumably resulting from microbial activity and pectin hydrolysis. In total, although the powder had excellent stability, the drink demonstrated obvious signs of degradation upon storage, highlighting the effects of storage conditions on both the types of jamun products [90].

## 5. Encapsulation of Jamun Extract

The bioavailability of natural food commodities has been a major concern in the food sector due to extreme processing conditions. To overcome the problem, encapsulation techniques have been commonly used to preserve sensitive food compounds. Encapsulation involves the entrapment of active ingredients inside a host cell, where active ingredients such as minerals, vitamins, and antioxidants are commonly preserved inside the core–shell, and the carrier wall used for encapsulation is commonly prepared from starch and its derivatives such as phospholipids, whey, and soya proteins. Some commonly used compounds for encapsulation are SA (sodium alginate), MD, and chitosan. MD, due to its low viscosity and sugar content, was commonly used for the encapsulation of Acn, whereas the presence of divalent cation and the hydrophilic nature of SA make it a good constituent for cell immobilization, and chitosan, obtained from the deacetylation process, was utilized for the core–shell manufacturing process due to its biodegradable and adhesive nature [91]. In a study, it was reported that a combination of 1.5% *w*/*v* SA with 0.8% *w*/*w* chitosan retained maximum Acn content (278.21 mg/100 mL) with an encapsulation efficiency of 12.4%, and the core–shell also prevented Acn degradation from 3.96% to 3.57% [92]. In a similar study, encapsulation of Acn with 1.5% SA and a core–shell of 2% guar gum combination enhanced encapsulation delivery efficiency by 95%. Polymerization of Acn and alteration of the negative charge present in SA by guar gum promoted a better release of cyanidin-3-glucoside Acn in the gastric fluid by 7.16 mg within a period of 1 h and 40 min and increased lightness, redness, and hardness value of jamun extract encapsulate by 51.63, 24.83, and 2.45 N [89]. Microencapsulation of Acn-rich jamun pulp (1:0.40–1:2, *w*/*w*) through ionic gelation and protein coating shows that with increasing the concentration of gelatin from 0% to 10% *w*/*w* in the coating solution, enhanced protein adsorption leads to a more robust protective layer. This process, however, results in a reduction in both moisture content (97.06–87.04 0.12%) and increased Acn levels ranging from 5.84% ± 0.62% to 0.78% ± 0.14%. As the protein content increases, the solids content of the microparticles also rises, which can dilute the concentration of Acn pigments due to the higher total solids content [93]. Encapsulation of jamun extracts was also utilized as a functional ingredient in the development of value-added chewing gum. Jamun extract (5 g/100 g) encapsulated using a SA and guar gum mixture at a 20:80 ratio through vibrating dripping extrusion resulted in the highest dissolution of phenolics (3.03–7.05 mg/g) within the first 10 min of chewing, which helps to inhibit oral infections. The release of phenolic compounds may result from the solubilization of sorbitol in the oral environment, the softening of the gum base, and the structural changes of the encapsulating polymer during simulated oral digestion [94]. Nanoagriculture represents a promising approach to improving soil health and enhancing plant growth through the use of environmentally friendly nanoparticles. The green synthesis of titanium dioxide nanoparticles from jamun has demonstrated nematode-toxic properties against *Meloidogyne incognita*, offering a safer alternative to conventional chemical nematicides without causing harm to plants. *Meloidogyne incognita* is a detrimental biotrophic parasite that significantly impacts agricultural productivity; the effective use of titanium dioxide at concentrations of 125 ppm shows potential for reducing juvenile mortality and inhibiting egg hatching [95].

Nanoencapsulation enhances drug delivery and prevents thermal degradation and oxidation of bioactive components. Spray drying with long residence time and short temperature confirms to be the best processing technique for nanoencapsulation of heat-sensitive compounds like Acns and polyphenols. Some limiting factors of the spray drying process are low solubility, hygroscopic and sticky nature of spray-dried powder, and to overcome these problems, MD and gum acacia can be used for encapsulation. As stated in a study, the addition of GA in spray-dried jamun powder increased TPC in jamun powder by 15.93 mg GAE/mL compared to the control sample, and the increase in TPC might be due to the preventive action of glycoproteins in GA against thermal degradation [96]. Temperature also plays a significant role in the spray drying process. If the drying rate increases during spray drying, evaporation will occur at a much faster rate, and the crust formed at the surface of the particle under such treatment conditions would be hard, which in turn will affect the shrinkage and size reduction of the particles. If the bulk density of the particles decreases, more air will get entrapped between the particles, and this, in turn, will have a negative effect on Acn stability, as reported by [97]. The incorporation of 25% MD in the core material increased powder yield up to 8.25%. MD also helps in preventing cluster formation of powder. Essential oils extracted from jamun leaves, such as L-*α*-terpineol and 2-methylene-4,8,8-trimethyl-4-vinyl, were also nanoencapsulated using nanoparticles prepared by the oxidation and precipitation of magnesium nitrite and barium chloride. The crystalline fraction of Mg-incorporated nanoparticles helped in the maximum retention of TPC (93.57 mg/GAE/L) and TFC (1540.55 mg/L).

Jamun was also found useful in nanoagriculture, representing a promising approach to improving soil health and enhancing plant growth through the use of environmentally friendly nanoparticles. The green synthesis of titanium dioxide nanoparticles from jamun has demonstrated nematode-toxic properties against *Meloidogyne incognita*, offering a safer alternative to conventional chemical nematicides without causing harm to plants. *Meloidogyne incognita* is a detrimental biotrophic parasite that significantly impacts agricultural productivity; the effective use of titanium dioxide at concentrations of 125 ppm shows potential for reducing juvenile mortality and inhibiting egg hatching [95].

## 6. Potential Applications in the Food Industry

The valorization of underutilized, nutritionally rich byproducts of the food processing industry has been providing new ways for unlocking their potential in the functional food industry or therapeutic food formulations. The term “nutraceutical,” coined in 1989 by Dr. Stephen L. DeFelice, blends the concepts of “nutrition” and “pharmaceutical,” referring to substances derived from foods that, while resembling pharmaceuticals, offer potential health benefits. Japan's 1991 introduction of Foods for Specified Health Use marked the first regulatory framework for functional foods, fostering the development of products enriched with beneficial additives [23, 98]. The global nutraceutical market, historically dominated by the United States, Japan, and Europe, has seen these regions near saturation, prompting a shift toward emerging markets like India. Although India represented just 2% of the global market in 2017, its nutraceutical industry grew to $11 billion by 2023, achieving a compound annual growth rate (CAGR) of 21% and is projected to capture 3.5% of the global market share [99]. India's burgeoning market features prominent local companies alongside global leaders, all operating within a regulatory framework established by the Food Safety and Standards Authority of India. This framework ensures the safety and quality of nutraceutical products through stringent standards [100]. To date, different research work has been focused on the extraction of bioactive compounds from jamun pulp, seed, kernel, and leaf portion and its utilization in the production of jamun-based/jamun-infused food products such as fruit/vegetable juice, wine, dairy products, bakery, and confectionery products (Figure 5) as mentioned below to improve the nutritional, organoleptic, and sensorial profile of the final food products. Also, Table 3 constitutes the application of jamun in different food products.

### 6.1. Jamun Pulp/Fruit Powder–Based Fruit Juice

Vegetable juices like jamun are also well known for their therapeutic properties, but due to the higher pH value, vegetable juices are easily susceptible to microbial attack. Furthermore, the juices are colorless and flavorless, so the blending of jamun juice with vegetable/any exotic fruit juices helps to overcome the high price of food commodities, improve shelf life, and also improve the aesthetic properties of the final beverage [108]. A similar study was reported by [18]. Ash gourd–jamun and bottle gourd–jamun blends increased the bioactive composition of juice. However, the former juice blend exhibited a modest reduction in TPC, Acn, DPPH, and inflammatory activity by 35%, 73%, 34%, and 35%, respectively, during storage. In comparison, the bottle gourd–jamun juice blend showed a lesser decrease in these parameters, which indicates the potential of the juice blend in functional foods. Jamun pulp, being very perishable and a seasonal fruit, is at times supplemented in powder form in fruit/vegetable juices and in dried fruit powder; with reduced volume, some economic benefits are also attained, such as being easy to handle, transport, package, and, most importantly, the availability of jamun in off-seasons as well. In fruit powder, the drying technique also plays a crucial role in the composition of the final product, as studied by [101] where freeze-dried jamun-infused pear juice had minimal degradation of Acn and phenolic due to water availability at the time of freezing. Further, during storage, the inversion of nonreducing sugars to RS (reducing sugar) increased RS content, and due to the acid hydrolysis of sugars to monosaccharides, TS (total sugar) content was reduced by 4.63% in the final product. A similar study was done by [102] where jamun syrup refrigerated at 4°C–7°C exhibited slower auto-oxidation of jamun Acn than the syrup stored at ambient room temperature 18°C–22°C. The stability of Acn during storage depends on factors such as the methoxy group, attached sugars present in Acn, and the presence of oxidoreductase like polyphenol oxidase, which increases the rate of Acn degradation during storage. In jamun/jamun-enriched fruit juices, the decline in ascorbic acid content might be due to the conversion of L-ascorbic acid to dehydroascorbic acid, and further degradation of ascorbic acid tends to increase the acidity of fruit juice due to the interaction of organic acids present in juice [109]. In a similar study, jamun (80%) juice blended with avocado (15%) and nannari (5%) improved ascorbic acid (23.76 mg/100 g), Acn (23.71 mg/100 mL), and AA (49.33%) of juice blend. A slight decline in ascorbic acid during storage might be due to the catalytic activity of fructose present in jamun [110].

### 6.2. Bakery and Confectionary

Bakery product nowadays is an important part of the human diet across the world, and there is no dispute that baked products are wholesome food products rich in carbohydrates, fats, sugar, and gluten. Moreover, the addition of jamun in baked products such as rice muffins not only aids in the preparation of antioxidant-rich gluten-free muffins but also improves the viscoelastic properties, that is, elasticity, viscosity, and rheology of the batter, which might be due to the action of dietary fibers making water unavailable in the batter (Table 3). Furthermore, with an increase in jamun pulp concentration, muffins turned slightly greenish, which could be utilized as an alternative to synthetic colorants in the bakery industry. The color of the crust portion of the muffin depends on chemical reactions such as the Maillard reaction and caramelization reaction, and together with this, RS (fructose) present in jamun also increases the rate of reaction [103]. In the future, jamun seeds could also be utilized for biscuit production at the commercial level. Wheat flour, the major ingredient in the biscuit industry, is deficient in dietary fiber, so the addition of jamun seed powder will be nutritionally advantageous. The type of drying techniques used for fruit juice/seed processing also plays a significant effect on the quality of the final product, as reported by [101] where chapattis prepared with freeze-dried jamun powder showed a 24.20% and 33.21% increase in TPC and AA, and no significant effect on puffing of chapattis was observed. The high hygroscopicity and adsorption capacity of freeze-dried jamun powder resulted in softer chapattis.

Confectionary products like baked products rich in sugar and carbohydrates have a higher caloric value that is not suitable for diabetic and obese people. To overcome this problem, Reference [59] developed a sugar-free jamun-based confectionary with a low calorific value (1.80 kcal/g) and glycemic index (GI) (48.55). As mentioned in Table 3, the confectionary is rich in dietary fiber, and the polymeric structure built by the dietary fibers cannot be easily attacked, which might be one probable reason for the low GI value. The confectionary also had a prebiotic score of 2.16, which can be attributed to the presence of polydextrin in the confectionary formulation, which promotes the growth of healthy bacteria, namely, *Bifidobacterium* and *Lactobacillus*, in the gut and improves resistance to invading pathogens. In addition, jamun can be used as a biocolorant in the food industry. Biocolorants are generally prepared from plant origin and are a good source of AA and could be a possible replacement for synthetic colorants. One such biocolorant, that is, Acn, extracted from jamun improved the DDPH scavenging activity from 25.06% to 98% and reduced water activity (*w*_a_) of marshmallow candy from 0.806 to 0.618 (Table 3). Furthermore, the IC_50_ value of Acn extract, that is, 3.60 mg/mL, was equivalent to the BHT IC_50_ value of 139.5 ppm, which indicates that the AA of jamun extract had better inhibitory activity than that of synthetic antioxidant [104] making it a good fit for consumption.

### 6.3. Wine

Jamun wine is a sparkling pink/purplish color fermented product with a pleasant effervescence. Organoleptic properties of jamun wine are mainly dependent on phenols imparting astringency and bitterness in wine, whereas the stable color of wine might be due to the intermolecular and intramolecular interaction of Acn resulting in pigmentation and volatilization of bioactive compounds of the final product, which in turn increases the nutraceutical properties of the wine prepared. Moreover, dilution and yeast strain play an important role in the production of good-quality wine, as reported by [111]. Optimal dilution was also found to be important for maintaining aerobic environmental conditions for yeast growth. In addition, yeast activity is inversely proportional to TSS which further decreases upon increasing fermentation time due to the inhibitory action of ethyl alcohol [105]. Consequently, a study carried out by [106] by two native isolates *Saccharomyces cerevisiae* and *Pichia gummiguttae* on the vinification process of jamun wine reported that TSS and bioactive composition of jamun wine slightly reduced after aging; this decline in TSS might be due to the utilization of residual sugars during fermentation or due to the oxidation of oligosaccharides with polyphenols, whereas oxidative degradation and condensation reaction of Acn with other phenolic compounds might have reduced Acn content. Furthermore, it was found that after aging, phenolic content was more prominent in *Saccharomyces cerevisiae* fermented wine than in the later isolate, which might be due to the presence of polysaccharide in *P. gummiguttae*. Similarly, ethanol concentration slightly reduces in wine after maturation, which might be due to the reaction between alcohol and acids resulting in ester formation. Esters are also produced from ethanol due to the reaction with short-chain fatty acid precursors [111]. Reference [106] reported that jamun wine is also free from browning reaction, but in the case of jamun seed–infused wine, polyphenols and polysaccharides leach out from the seed portion and later form a complex with the polyphenols, leading to the browning reaction of jamun wine.

### 6.4. Colorant and Natural Dye

In accordance with the worldwide interest toward healthier foods, the market for food colorants is giving rise to the search for natural alternatives to replace artificial food colors. Berries have potential market interest due to their high content of bioactive compounds that provide AA and are known to confer many health benefits like anti-inflammatory, immunomodulatory, antimicrobial, improvement of vision, and neuroprotective effects [72]. Jamun has also proven to be an effective natural dye for use as a photosensitizer in dye-sensitive solar cells. The polyphenolic compounds, particularly Acn present in jamun, exhibit strong anchoring groups that effectively bind to titanium dioxide electrodes, facilitating efficient charge transfer, significantly boosting the efficiency of solar cells. Among the tested natural dyes, jamun achieved the highest efficiency at 1.09%, outperforming those made with black plum and blackberry, which had efficiencies of 0.55% and 0.38%, respectively [112]. Blackcurrant juice extracted by enzymatic maceration under optimal conditions (200 ppm enzyme, 45°C, and 60 min) improved a 2.4-fold increment for TPC and 2.8-fold for Acn along with a 15% increase in juice yield. An attractive color product was obtained, also containing blackcurrant bioactive compounds (0.56 ± 0.03 mg GA/g, 0.20 ± 0.02 mg cyd-3-glu/g, and 0.16 ± 0.02 mg of GA/g for TPC, ACs, and AC, respectively). This jelly was stable regarding syneresis and antioxidant compounds for 15 days at 4°C. The developed blackcurrant powder could be useful as a natural colorant and source of bioactive compounds for several food applications [72].

### 6.5. Other Products

Whey, a byproduct of acid-treated milk, is renowned for its high protein, lactose, and nutrient content. Despite its perishable nature, whey holds promise as a functional food ingredient. In a study, whey utilized in the creation of innovative food products, that is, whey and jamun-infused ice popsicles, exhibited a notable 60% *α*-amylase inhibitory activity, potentially attributed to the presence of phytochemicals such as gallic acid and catechins in jamun. The inhibitory effect of *α*-amylase was also confirmed through an in vivo study where the initial glucose level in hyperglycemic mice was 110.5 mg/dL, and after the oral intervention of 2, the glucose level in mice fed with a regular diet and a jamun-fortified diet was 177.75 and 139.25 mg/mL, indicating the popsicles to be a better fit for diabetic patients [2]. Jamun juice could also be used for yogurt and fermented milk production; as reported by [113], addition of jamun in yogurt reduced fat content from 3.52% to 3.17%, which might be due to the low total solid content of jamun, and with increasing jamun concentration, acids present in jamun fastened the fermentation reaction and increased acidity in yogurt. Together with this, sugar present in jamun, like fructose, also increased the TS content of yogurt from 6.68% to 7.04%, promoting the production of a low-fat and sweetened yogurt product. In the food industry, the underutilized pomace portion of jamun could also be used as a low-cost substitute for functional food development, and one such product, that is, jamun pomace powder–enriched ice cream, was developed by [20] where jamun pomace not only reduced overrun from 61.19% to 48.66% but also improved ice cream hardness from 21.88 to 34.26. Fiber present in pomace powder not only improved the ice cream hardness by increasing TSS but also helped to reduce ice cream mix fat content from 11.5% to 9.40%. The fibers present in jamun pomace effectively improved final product quality by increasing the water retention capacity of the ice cream mix, which in turn increased the melting point of the ice cream.

Jamun could also be used for jam preparation due to its various health-promoting factors (Table 3). In a study, it was reported that mixing jamun pulp with apple and kiwi pulp promoted better release of tartaric acid (25.28 and 26.24 mg/100 g), ascorbic acid (0.02–0.08 mg/100 g), and lactic acid (11.02 and 23.95 mg/100 g) into the fruit mix jam, and in the realm of food coloration, jamun-based jams exhibited a vibrant red hue, contrasting with apple and kiwi-infused variants which demonstrated a 10% and 7.5% reduction in color saturation, respectively, compared to the control sample [107]. Jamun-based jams are also reported to have high phenolic content (5.58 mg GAE/g) and DPPH scavenging activity (47.97%) and low fat (0.15%) and sugar (69.12%) in comparison to fruit jams made out of peach, apple, pineapple, and mango, and most importantly, the viscosity of jam (500.3 poise) was found to be similar to jams available on a commercial scale. The viscosity of a jam decreases when force per unit area increases under controlled shear, and with increasing viscosity, the spreadability of the jam also increases [114].

### 6.6. Packaging

Food packaging plays a crucial role both in attracting consumers and providing important information about the quality and safety of processed fruit products. Among various packaging materials, glass is particularly effective due to its impermeability to gases and vapors, which helps preserve the freshness, flavor, and nutritional content of food over time [115]. It was stated that glass packaging for jamun has maintained higher levels of DPPH assay activity (21.75%), ascorbic acid (10.48 mg/100 mL), and other quality indicators such as TSS and titratable acidity compared to polythene and plastic packaging. Polythene packaging has a weak protection against light and oxidation, leading to a greater loss of vitamin C. Thus, choosing the right packaging material is essential to ensure the preservation of food quality and safety [116]. It was stated that immersing fully ripe jamun fruits in a 1.5% chitosan solution combined with 1.5 mM salicylic acid significantly enhances the storage quality of jamun by reducing the weight loss by 19.5% and decay loss by 49.9% compared to control fruits. Additionally, it improved the retention of Acn and TPC by 1.34 to 1.69 times over a 6-day storage period. The combined treatment proves to be an effective coating method for extending the shelf life of jamun at ambient conditions [117]. Similarly, jamun treated with a 1.0 mM concentration of putrescine through the immersion method significantly enhances their storage quality under ambient storage conditions (27°C, 85% RH) for 6 days. The putrescine-treated fruits exhibited a notable reduction in weight loss and spoilage, achieving a 1.5-fold improvement compared to the untreated control [118].

The valorization of agricultural byproducts like jamun seeds represents a promising approach to developing sustainable plastic alternatives. Repurposing waste materials helps to conserve biodiversity and advance a circular economy [119]. In a recent study, bionanocomposite packaging films were developed utilizing agricultural byproducts such as jamun seed starch and tamarind kernel xyloglucan. It was reported in a study that jamun skin–infused methylcellulose films were biodegraded in the soil after 15 days. In contrast, petroleum-based plastic packaging requires 100 years to decompose in the soil, causing a serious environmental threat [120]. Further, jamun seed portions and xyloglucan are reported to form a dense polymer network that improves the overall packaging properties of the films. Notably, adding 3% *w*/*w* chitosan nanoparticles significantly increased tensile strength (20.42 MPa), elastic modulus (0.8 GPa), and contact angle (89°), while reducing the water vapor transmission rate (13.26 g/h·m^2^) [119].

## 7. Challenges

Jamun, a lesser-known fruit often categorized as minor due to its limited palatability, is predominantly cultivated in an unstructured manner on wastelands and along roadsides. Jamun cultivation is feasible in adverse conditions such as arid, semiarid, marshy, and wasteland regions where other crops fail. It is of both economic and medicinal importance, making a significant contribution toward India's fruit yield, with Odisha alone contributing 2.6% in 2019–2020. But its production is inhibited by inefficient and labor-intensive harvesting that causes considerable postharvest loss and quality deterioration of fruit, discouraging many farmers from cultivating it, and hence, this affects the marketing of jamun. In India, however, there is a notable absence of organized farming practices for this fruit. Despite the significant nutritional and therapeutic value of jamun, a considerable portion of the harvest, around 0.5 million tons in India alone, is lost due to its high perishability and short storage life of just 2–3 days at ambient temperatures, which makes distribution challenging, particularly where access to faraway markets is restricted by cost and logistics. There is an urgent need to develop effective methods to extend the postharvest life of fruit under these conditions [117, 118]. This highlights the need for increased awareness and development of structured cultivation methods to enhance its marketability and economic value [121]. Conventional packaging techniques do provide some protection, such as modified-atmosphere packaging that can add up to 23 days of shelf life under controlled storage conditions. Resolution of these problems through mechanical harvesting and improved postharvest handling, storage, and packaging is critical in minimizing losses and improving the market viability of the crop [122, 123]. Mathematical modeling and artificial intelligence (AI)–based simulations offer significant advantages in optimizing berry processing techniques such as drying, disinfecting, and freezing of berries. By integrating AI models with traditional detection data, these technologies enable rapid, nondestructive testing of product quality. However, the lack of advanced detection equipment in the industry remains a critical issue that requires further innovation and collaboration between manufacturers and researchers to develop innovative, rapid, and nondestructive tools [124].

## 8. Conclusion

The multifaceted attributes of jamun, from its antioxidant-rich pulp to its beneficial leaves and seeds, position it as a promising functional food ingredient. On a commercial scale, the underutilized fruit also presents a significant opportunity for addressing both nutritional needs and economic viability. However, current practices result in substantial annual losses of 20%–30% of jamun in postharvest, leading to missed opportunities, especially in meeting the dietary needs of vulnerable populations like pregnant, lactating mothers and infants. In the food processing sector, addressing these losses through different conventional and green processing techniques could unlock the nutritional value of jamun, especially crucial for the vulnerable population. Encapsulation further ensures preservation, creating possibilities for their integration into functional foods. The substantial pomace portion generated after jamun processing also becomes a cause for environmental challenges such as oxide emission and groundwater contamination. But today, scientists advocate for utilizing the pomace portion, recognizing its rich composition of fibers, antioxidants, phenols, and other nutrients. Repurposing this byproduct into low-calorie, low-fat functional food products would help gain consumers' trust in the competitive food sector. In the food processing industry, the technique of blending stands out as a pivotal method in enhancing the quality of processed food products by infusing jamun extract or pomace with different food entities, creating diverse flavors, aromas, textures, and tastes to cater to various preferences. In essence, tapping into the underutilized potential of jamun, both in terms of its fruit and byproducts, not only addresses nutritional deficiencies but also presents an opportunity to create sustainable and appealing food products while mitigating environmental impact. As consumer preferences continue to evolve toward healthier and eco-friendly choices, jamun stands out as an underutilized resource with immense potential.

## Figures and Tables

**Table 1:** Nutritional composition of jamun pulp and seed portion.

Moisture (mg/100 g)	76.1–90.6	54.9–65.7
Ash (g %)	0.93–1.03	0.98–1.01
Total sugar (mg/g)	6.51–17.6	1.03–2.16
Protein (mg %)	3.16–5.95	4.72–7.14
Crude fiber (g %)	2.95–3.65	6.17–10.5
Free amino acid (mg %)	7.54–18.9	4.84–9.90
Starch (g %)	15.1–22.4	22.8–29.4
Titratable acidity (%)	0.8–2	ND
Ascorbic acid (mg %)	66–137	90–137
Total phenol (mg GAE/100 g)	221.83–1348	4500–5670
Total flavonoid (mg QE/g)	29.45–36	29.45–208
Total Acn (mg %)	29.7–47.7	ND

*Note:* Jan et al. [2], Ahmed et al. [13], Gajera et al. [14], Singh et al. [9, 10], Coelho et al. [15], Shrikanta et al. [16], Branco et al. [17].

**Table 2:** Pharmacological characteristics (antidiabetic, antioxidant, anticancer, and antimicrobial activity) of jamun.

**Extraction technique**	**Bioactive compounds**	**Pharmacological activity**	**Key findings**	**Assay**	**References**

		**Antidiabetic activity**			
Cold extraction	Alkaloids, caffeine acid, tannin, quercetin	IC_50_ (methanolic extract) 129.98 *μ*g IC_50_ (chloroform extract) 430 *μ*g IC_50_ (ethyl extract) 702.24 *μ*g IC_50_ (acarbose) 879.99 *μ*g	Phytochemicals inhibit pancreatic *α*-amylase and prevent prandial hyperglycemia by preventing the breakdown of starch into sugar	In vitro	Rauf et al. [43]
Batch extraction	Catechins, gallic acid	IC_50_ (kernel extract) 9.03 *μ*g/mL IC_50_ (acarbose extract) 23.95 *μ*g/mL	Epi form and galloyl moiety of catechins decelerate saccharification process and control hyperglycemia	In vitro	Mahindrakar et al. [49]
Soxhlet extraction	Ellagic acid, chlorogenic acid	IC_50_ (methanolic extract) 8.3 *μ*g/mL IC_50_ (acarbose extract) 24.7 *μ*g/mL	Phenols have better *α*-amylase inhibition rate than standard acarbose	In vitro	Gajera et al. [14]
Solvent extraction	Gallic acid, quercetin, triterpenoid	Fasting hyperglycemia reduced by 27.4%	Triterpenoids maslinic and oleanolic acid decreased *α*-glucosidase and *α*-amylase activity in the small intestine	In vivo	Xu et al. [48], Li et al. [47], Raza et al. [50]
		**Antioxidant activity**			
Lyophilization	Flavonoids, stilbenes, coumarins	IC_50_ (seed extract) 0.40 mg/mL IC_50_ (pulp extract) 1.06 mg/mL	Bioactive compounds scavenge free radicals and inhibit scavenging of ROS during normal cell metabolism	In vitro	Shrikanta et al. [16]
Three-phase partitioning	Gallic acid, tannic acid, catechin	IC_50_ (kernel extract) 12.15 *μ*g/mL IC_50_ (gallic acid) 5.9 *μ*g/mL	Phenolic compounds inhibit the growth of ROS and nitrogen species responsible for oxidative degradation	In vitro	Mahidrakar and Rathod [49]
Fractional separation	Quinic acid, ellagic acid, caffeic acid	IC_50_ (extract) 81 *μ*g/mL IC_50_ (ascorbic acid) 75 *μ*g/mL IC_50_ (quercetin) 68 *μ*g/mL	Antioxidant activity of crude extract and its fractions is associated with hydrogen donating ability	In vivo	Qamar et al. [51]
Hot percolation	Quercetin, gallic acid, ferulic acid	80.4%–94.8% inhibition 66.7%–83.7% inhibition (Kernel and pulp portion)	Radical scavenging activity directly proportional to the phenolic composition of jamun	In vitro	Gajera et al. [52]

		**Anticancer activity**			
**Extraction technique**	**Model**	**Bioactive compounds**	**Mechanism**	**Assay**	

	Breast and colon cancer cells	Ellagic acid, luteolin, kaempferol, Acn, isorhamnetin	Bioactive compounds promote the induction of metabolizing enzymes, regulate gene expression, modulate cell proliferation and apoptosis	In vivo	Charepalli et al. [53], Aqil et al. [54]
	Embryonic kidney cells, breast, ovary, lungs, and prostate gland cancer cells	Acn, ferulic acid, caffeic acid, and gallic acid	Phenolic compounds enhance antiproliferation and cytotoxic activity by inhibiting growth of mutated, neoplastic, and preneoplastic cells responsible for cell cancer	In vitro	Gibbert et al. [55], Sharma et al. [56], Yadav et al. [57]

		**Antimicrobial activity**			
**Extraction technique**	**Pathogenic strain**	**Bioactive compound**	**Mechanism**	**Assay**	

Steam distillation	*Leishmania amazonensis*	Essential oil, *α*-pinene	Essential oil due to its lipophilic property passes through the cytoplasmic membrane and interacts with membrane components, changing the membrane permeability and promoting cell lysis	In vivo	Rodrigues et al. [58]
Solvent extraction	*Vibrio parahaemolyticus*	Vitamins C and E	Vitamins provide better stress resistance and increase specific and cell-mediated immunity against microbial infection	In vivo	Prabhu et al. [3]
Freeze drying	*Staphylococcus aureus*, *Escherichia coli*	Gallic acid, caffeic acid, sinapic acid, quercetin	Chelation of flavonoids and phenolic acid increases the death rate of microorganisms	In vitro	Singh et al. [9, 10]
Hydrodistillation	*Rhizoctonia solanine* *Choanephora cucurbitarum*	*α*-Terpineol, 7-hydroxycalamenene, 1-epi-cubenol, 7-acetoxy calamenene	Low molecular weight alcohols, terpineol exhibits better antifungal due to the presence of lesser carbon content which promotes easy vaporization in the volatile phase	In vitro	Saroj et al. [11]

**Table 3:** Applications of jamun in the food industry.

**Food product**	**Product formulation**	**Chemical composition**	**Key findings**	**References**
Juices				
Ash gourd (*Benincasa hispida*) + jamun blend	Juice blend proportion 35:20	TSS 9.5^0^B TPC 267.45 *μ*g GAE/mL Acn 8.85 *μ*g C3GE/mL DPPH assay 1727.2 *μ*g AAE/mL	TPC and DPPH scavenging activity was directly proportional to juice concentration During storage, TSS of juice ↑ due to break down of polysaccharides to mono and oligosaccharides	Palamthodi et al. [18]
Jamun powder + pear juice (*Pyrus* species)	Pear juice + 1%–5% jamun powder (−60°C)	TSS 14–14.20^0^B TS 9.93–10.39 mg/100 g RS 7.98–9.81 mg/100 g TPC 120.37–109.59 mg/100 g Acn 6.89–2.17 mg/100 g AA 75.21%–82.63%	The availability of liquids during freeze drying minimized the degradation loss of bioactive compounds	Kapoor and Ranote [101]
Syrup	Juice 25%–40% TSS 65, 70^0^B	TPC 79.32–117.25 mg/100 mL Acn 41.50–69.24 mg/100 mL AA 20.45%–29.32%	Interaction between phenols and proteins contributed to the gradual ↓ in syrup quality over the storage period	Bhatt et al. [102]
Bakery and confectionary				
Jamun-supplemented muffins	Rice flour + jamun (0–30 g) + water (64–85 mL)	*G*′ 512–2734 Pa *G*^″^ 64.13–298.58 Pa Tan 0.21–0.37 tan TPC 496.5–637.9 *μ*g GAE/g	With ↑ fruit part viscoelastic properties: Storage module and loss module ↑ whereas tan *δ* indicates batter rheology↓ The lower the tan *δ* value, the better the elasticity property of the batter	Singh et al. [103]
Jamun-enriched chapattis	FD (−30°C, 60 h) jamun powder (1%–5%) + wheat flour + water	Water absorption 67.26% TPC 108.79 mg GAE/100 g Acn 4.18 mg/100 g AA 83.99%	Acn is highly sensitive to heat treatments but in FD jamun powder the absence of liquid during drying minimized Acn degradation	Kapoor et al. [101]
Jamun-based confectionary	Jamun pulp (−18°C) + jamun seed powder (60°C, 24 h) + hydrocolloids	TPC 3.72 mg GAE/100 g DPPH 0.98 mg BHA/g Total dietary fiber 15.71 g/100 g Vitamin C 38.35 mg/100 g	Jamun confectionary has 53% lower calories than many commercially available sugar added fruit jellies According to European regulations, fruit candy with fiber content more than 6 g/100 g is considered a fiber-rich product	Sehwag and Das [59]
Marshmallow candy	AcnEE (3–7 g)/SC (0.10%) + marshmallow (100 g)	*a*_w_, control 0.84–0.69 *a*_w_, SC 0.80–0.66 *a*_w_, AcnEE 0.83–0.61	The moisture absorption and retention ability of AcnEE ↓ *a*_*w*_ of marshmallow candy ↓	El-Messiry et al. [104]
Wine				
Jamun seed + powder fortified wine	Juice (18^0^B) + seed and pulp powder (286.4, 300 mg/100 mL) Storage: 0–90 days	EI 9.94%–9.39% Tannin 1.63–1.39 mg/100 mL Acn 55.05–51.32 mg/100 mL	In fortified wines ethanolic content and in the control sample tannin significantly	Singh et al. [105]
Jamun wine	Pulp (2 kg) + inoculum (*Saccharomyces cerevisiae*) + fermentation (21 days 22°C)	Young and aged wine pH 3.42–3.78 TS 5.6 and 2.36 g/100 mL Alcohol 10.68% and 9.88% Acn 228 and 166 mg/L Tannins 1.01 and 0.72 *μ*g/mL	Acn ↓ due to the presence of nonoxidative conditions TS ↓ due to the precipitation of oligosaccharides/alternatively due to the fermentation of reducing sugars	Venugopal and Appaiah [106]
Other products				
Whey-based jamun ice popsicles	Juice (20–50 mL) + whey (50–80 mL)	TPC 7.89–5.96 mg GAE/mL TFC 4.58–3.56 mg QE/mL AA 78%–68%	Bioactive compounds due to their volatile nature might have undergone oxidative degradation during storage	Jan et al. [2]
Jamun pomace–flavored ice cream	Pomace (50°C, 32 h) + ice cream mix (10% fat, 11% SNF, 15% sugar, 0.2% stabilizer)	TSS 29.17–39.43^0^B TA 0.19%–0.52% Fat 11.15%–9.40% Fiber 0%–0.620% Hardness 21.88%–34.26% Overrun 61.19%–48.66%	In ice cream mix with ↑ pomace concentration air absorption ↓ during freezing which in turn ↑ overrun and hardness of the ice cream	Shelke et al. [20]
Mix fruit jam	Apple and kiwi pulp are added to jamun pulp in the ratio 1:4 + 250 g sugar + 0.2% pectin	pH 2.93–3.01 TSS 64.67–66.67^0^B MC 28.79%–35.89% TA 1.0%–1.64% Ascorbic acid 0.02–0.08 mg/100 g AA 39.15%–46.75% TPC 3.28–5.48 mg GAE/g	A slight difference in TSS and moisture content of jam might be due to the variation in fruit ripeness or cooking time Mixing of fruit pulps improved AA of the final product by increasing the concentration of ascorbic acid and phenolic content	Garg et al. [107]

*Note:* The symbols **+**, ↑, and ↓ stand for addition, increase, and decrease, respectively.

## Data Availability

All data of this study are available within the manuscript.

## Nomenclature

ATP: adenosine triphosphate
ROS: reactive oxygen species
MHz: megahertz
GHz: gigahertz
NTU: nephelometric turbidity unit
mg/kg: milligram per kilogram
MPa: megapascal
CE/g: cyanidin-3-glucose equivalent per gram
SI: selectivity index
US: ultrasonication
MVT: microwave treatment, complete
S^−1^: blocking constant
Sm^−2^: cake filtration constant
SA: sodium alginate
MD: maltodextrin
GAE: gallic acid equivalent
QE: quercetin equivalent
mg: milligram
g: gram
mg/100 g: milligrams per 100 g
mg/g: milligram per gram
TSS: total soluble solids
^0^B: degree brix
SC: synthetic color
AcnEE: anthocyanin-enriched extract
TPC: total phenolic content
AA: antioxidant activity
DPPH: 2,2-diphenyl-1-picrylhydrazyl
BHA: 3-tert-butyl-4-hydroxyanisole
QE: quercetin
TS: total sugar
RS: reducing sugar
TA: titratable acidity
*G*′: storage modulus
*G* ^″^: loss modulus
tan *δ*: rheology of batter
*a*_w_: water activity
EI: ethanol index
C3GE: cyanidin-3-glucoside equivalents
*μ*g/mL: microgram per milliliter
*μ*g/g: microgram per gram
g/100 g: gram per 100 g
g/100 mL: gram per 100 mL
mg/100 mL: milligram per 100 mL
mg/mL: milligram/milliliter
mg/g: milligram per gram
mg/L: milligram per liter
Pa: Pascal
